# Underutilisation of routinely collected data in the HIV programme in Zambia: a review of quantitatively analysed peer-reviewed articles

**DOI:** 10.1186/s12961-017-0221-9

**Published:** 2017-06-13

**Authors:** Tendai Munthali, Patrick Musonda, Paul Mee, Sehlulekile Gumede, Ab Schaap, Alwyn Mwinga, Caroline Phiri, Nathan Kapata, Charles Michelo, Jim Todd

**Affiliations:** 1 School of Public Health, University of Zambia, Lusaka, Zambia; 2 Ministry of Health, Lusaka, Zambia; 3 Department of Population Health, London School of Hygiene and Tropical Medicine, London, United Kingdom; 4 Zambia AIDS Related Tuberculosis (ZAMBART) Project, Lusaka, Zambia; 5 MeSH Consortium, Department of Social Economic and Health Research, Faculty of Public Health and Policy, London School of Hygiene and Tropical Medicine, London, United Kingdom

**Keywords:** Routinely collected data, HIV, Zambia

## Abstract

**Background:**

The extent to which routinely collected HIV data from Zambia has been used in peer-reviewed published articles remains unexplored. This paper is an analysis of peer-reviewed articles that utilised routinely collected HIV data from Zambia within six programme areas from 2004 to 2014.

**Methods:**

Articles on HIV, published in English, listed in the Directory of open access journals, African Journals Online, Google scholar, and PubMed were reviewed. Only articles from peer-reviewed journals, that utilised routinely collected data and included quantitative data analysis methods were included. Multi-country studies involving Zambia and another country, where the specific results for Zambia were not reported, as well as clinical trials and intervention studies that did not take place under routine care conditions were excluded, although community trials which referred patients to the routine clinics were included. Independent extraction was conducted using a predesigned data collection form. Pooled analysis was not possible due to diversity in topics reviewed.

**Results:**

A total of 69 articles were extracted for review. Of these, 7 were excluded. From the 62 articles reviewed, 39 focused on HIV treatment and retention in care, 15 addressed prevention of mother-to-child transmission, 4 assessed social behavioural change, and 4 reported on voluntary counselling and testing. In our search, no articles were found on condom programming or voluntary male medical circumcision. The most common outcome measures reported were CD4+ count, clinical failure or mortality. The population analysed was children in 13 articles, women in 16 articles, and both adult men and women in 33 articles.

**Conclusion:**

During the 10 year period of review, only 62 articles were published analysing routinely collected HIV data in Zambia. Serious consideration needs to be made to maximise the utility of routinely collected data, and to benefit from the funds and efforts to collect these data. This could be achieved with government support of operational research and publication of findings based on routinely collected Zambian HIV data.

## Background

Worldwide, many countries routinely collect data on HIV care and services, which is then used to provide national and international indicators about the HIV epidemic. These indicators provide information and insight to aid policymakers and planners when making important decisions about HIV services, to request for further research, and in advocacy for new initiatives and funding [1, 2].

Sub-Saharan Africa is home to approximately 71% of the people living with HIV [3]. Zambia is a high HIV burden country within sub-Saharan Africa, having a national HIV prevalence of 11.6% and almost 980,000 people living with HIV in 2016 [4–6]. The HIV epidemic in Zambia is generalised and is mainly attributed to unprotected heterosexual activity [7]. This creates a need to monitor the HIV epidemic by focusing on indicators of effective prevention and on the quality of the HIV services in Zambia [8]. Six key service areas are prioritised in the country for prevention and treatment of HIV. These are (1) voluntary medical male circumcision (VMMC); (2) condom programming; (3) behavioural change; (4) HIV testing and counselling (HTC); (5) prevention of mother-to-child transmission (PMTCT); and (6) treatment and retention in care [7]. At national level, these programmes are monitored using routinely collected data and periodic country representative surveys.

Routinely collected data can also be used to understand the effectiveness of services and to improve decision-making in the healthcare system. The benefits in using routinely collected data include wider coverage of recorded items from across the whole country and the longitudinal nature of the data allowing estimation of trends and changes in the use of services [9]. The use of these data in this way is cost effective, as it is already collected and readily available for analysis. Therefore, research can be conducted in a timely and cost-efficient manner [10]. It can be the basis of sampling for clinical trials, cohort studies and case-control studies, as matched case-control analyses can be performed repeatedly over long time periods [11]. Routinely collected data are usually clinic based, but results from analysis of such data can be generalisable to the whole population if the services are widely used and serve all sections of society [10]. One of the main data collection systems for the routine collection of data from the HIV programme is SmartCare, which is one of the largest electronic patient monitoring systems (PMS) in Africa and is used in South Africa and Ethiopia [12]. In Zambia, the SmartCare database is used as a PMS for HIV services, and the data are used to monitor and plan improvements in the country’s HIV programme. SmartCare has been used as a pilot since 2004, and was officially rolled out in 2006 [13], with 528 clinics using SmartCare in 2012, and implemented in more than 700 clinics that provide antiretroviral therapy services by 2013 [14, 15]. SmartCare data, in facilities where it is available, is used to provide aggregate reports for DHIS2, and other health management information systems at the district level. In some health facilities, the SmartCare data can inform the drug ordering and the laboratory information systems, but this is not possible in most health facilities in Zambia.

Research has revealed that countries like Zambia, with one of the highest HIV/AIDS prevalence rates in Africa, are not the largest contributors to research on HIV/AIDS. This was evident in a review of three journals focusing on HIV/AIDS [16], which showed that the United States of America and Western Europe accounted for 85% of all published articles between 1986 and 2003. In sub-Saharan Africa, 50% of all publications on Africa indexed in PubMed between 1981 and 2009 were from South Africa, Uganda and Kenya. Zambia was ranked seventh, with 922 publications within that period, translating to approximately 32 publications per year [17]. This paper is a review of published studies using routinely collected HIV data from Zambia from 2004 to 2015, within the six areas of focus (VMMC, condom programming, behavioural change, HTC, PMTCT, and treatment and retention in care). We sought to examine the extent to which routinely collected HIV data has been analysed quantitatively for publication and identify gaps that exist across the six prioritised areas. It is hoped that findings from this review will potentially inform guidelines and strategies as well as stimulate policy dialogue in the use of routinely collected data.

## Methods

### Literature search strategy

We conducted a literature review of studies that reported results from routinely collected HIV data in Zambia. We utilised a detailed search protocol and standard systematic review procedures (Additional file 1) for papers which utilised routinely collected HIV data from primary to tertiary healthcare settings, using SmartCare or other electronic or paper-based PMS data in Zambia. We included studies published between 2004 (when SmartCare started) and November 2015. The search was conducted between July and November 2015. We selected only original articles from peer-reviewed journals on HIV studies conducted in Zambia utilising routinely collected data and quantitative methods of data analysis. All reported studies relevant to our search topic were reviewed, regardless of sample size. Articles were excluded if they were not written in English or where the specific results for Zambia were not reported from regional or multi-country studies. Clinical trials and intervention studies that did not take place under routine care conditions were also excluded, although community trials that referred patients to the routine clinics were included.

We searched the PubMed, Google Scholar, Directory of Open Access Journals, and African Journals Online databases for articles on HIV in Zambia that utilised routinely collected data (Table 1). We used a combination of search words that included “HIV”, “SmartCare” and “routinely collected data”, among others (Table 1). One of the authors (TM) searched for articles and extracted the data from included studies, while another author (SG) reviewed the extracted data for discrepancies. All discrepancies were discussed and resolved. A standard data extraction form was used to review and extract data such as sample size, study design, number of study sites, dates of data collection, year of publication and main outcomes.

**Table 1:** Search strategy

Database	Search terms
Google Scholar (July 3, 2015)	HIV + SmartCare + Zambia HIV + routine + data HIV/AIDS + “routinely collected data” + Zambia Condom + HIV + Zambia
PubMed (July 15, 2015)	HIV/AIDS + “routinely collected data” + Zambia + routine data Condom + HIV
African Journals Online (November 6, 2015)	HIV + routine + data + Zambia + condom + "routinely collected data"﻿ + SmartCare
Directory of Open Access Journals (November 11, 2015)	HIV + Zambia + condom + "routinely collected data﻿" + SmartCare

### Data analysis

The selected papers could only be categorised by the six programme areas as the range of topics covered prevented aggregated statistical analysis of findings. All eligible articles were further grouped by the populations used in the papers, namely adult (males and females above 15 years of age), women and children (under the age of 15 years) to assess how effectively the priority areas cover the different age categories. The eligible articles were also analysed based on institutions that collaborated to publish the articles.

## Results

**Fig. 1:**
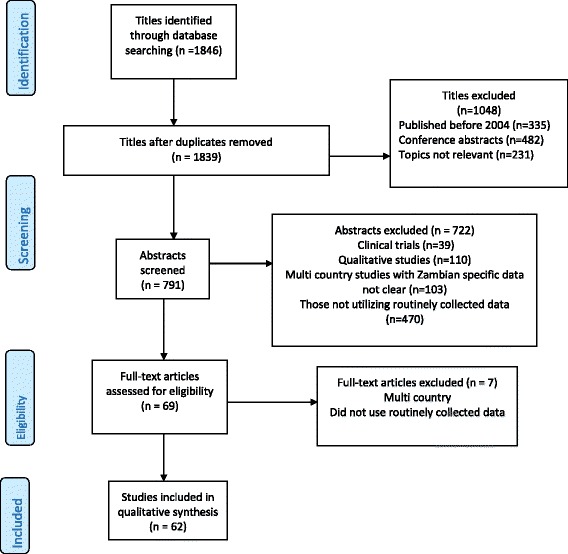
Flow diagram of review of routinely collected HIV data in Zambia

A total of 1846 titles were reviewed and 1048 were excluded because they were not published in journals (n = 482), were published before 2004 (n = 335), or the topics were not relevant (n = 231). A total of 791 abstracts were then reviewed. Of these, some were excluded because they were clinical trials (n = 39), qualitative studies (n = 110), or did not use routinely collected data (n = 470), or were multi-country studies that did not include specific data on Zambia (n = 103) (Fig. 1). From these, 69 full length articles were selected, of which seven were found to be multi-country studies that did not use routinely collected data, and the remaining 62 were considered for categorisation. The articles were then classified into the six HIV service areas.

Overall, 15 articles addressed PMTCT, four focussed on HTC, four covered social and behavioural change (Table 2), and 39 covered treatment and retention in care. Our search did not reveal any articles that used routinely collected HIV data in Zambia reporting outcomes in the areas of condom programming or VMMC utilising quantitative methods.

**Table 2:** Studies utilising routinely collected data in HIV testing and counselling, prevention of mother-to-child transmission (PMTCT) and social behavioural change programmes in Zambia

First author, year of publication	Data collection period	Sample size	Sampled population	Outcomes assessed
HIV testing and counselling				
Topp et al. [31]	2008–2011	2239	Adults only	CD4 count, haemoglobin level, BMI, education level, partner’s HIV status
Topp et al. [32]	2008–2010	44,420	Adults only	HIV testing, enrolment into care
Czaicki et al. [33]	2011–2012	10,806	Adult couples	Cohabitation length, prior HIV testing, current antiretroviral use
Kankasa et al. [34]	2006–2007	15,670	Children	HIV testing, testing coverage, HIV counselling,
PMTCT				
Killam et al. [35]	2007–2008	13,917	Women initiating ART	Women eligible for ART, women initiating ART
Stringer et al. [36]	2001	17,263	Women only	Women tested, mothers and babies receiving NVP
Stringer et al. [37]	2003	8787	Mother baby pairs	Gravidity, offered testing, tested, infant given NVP
Chibwesha et al. [38]	2007–2010	1813	Mother baby pairs	CD4 count, date of highly active ART initiation, infant HIV status
Liu et al. [39]	2007– 2009	13,888	Women initiating ART	CD4 count, haemoglobin level, syphilis, tuberculosis, HIV status
Chintu et al. [40]	2004–2006	6740	Women on ART	Mortality, NVP exposure, CD4 count, haemoglobin level
Mandala et al. [41]	2007–2008	14,815	Women on ART	CD4 count, initiated on ART
Torpey et al. [20]	2005–2008	9723	Women on ART	HIV testing, enrolled to care, received ART
Chibwesha et al. [42]	2009–2010	18,407	Women initiating ART	CD4 count, haemoglobin level, use of contraceptives
Stringer et al. [43]	2002–2006	243,302	Women and baby pairs	HIV status, number testing positive, attended antenatal care
Mulindwa [44]	Not stated	146	Women initiating ART	NVP toxicity, hepatic toxicity, WHO grading of toxicity
Ngoma et al. [45]	2008–2009	279	Women only	HIV-free at 12 months, mortality rates, HIV transmission
Torpey et al. [46]	2007–2010	28,320	Children only	HIV testing, type of PMTCT regimen
Torpey et al. [47]	2007–2009	8237	Children	HIV testing, type of PMTCT regimen
Albrecht et al. [48]	2001–2003	760	Women only	PMTCT drug adherence, partner disclosure
Social and behavioural change				
Kankasa et al. [34]	2004–2007	27,115	Adults on ART	Adherence, mortality, loss to follow-up, CD4 count
Goldman et al. [49]	2006–2007	913	Adults on ART	Adherence, viral load
Carlucci et al. [50]	2006	542	Adults on ART	Drug adherence
Birbeck et al. [51]	2005–2006	255	Adults on ART	Drug adherence

*ART* antiretroviral therapy, *BMI* body mass index, *NVP* nevirapine

The majority of the papers were mostly large samples, with thousands of subjects, covering many different health facilities. The articles on HIV treatment and retention in care covered topics such as enrolment and retention into antiretroviral therapy, effectiveness of different drug regimens, coinfections with laboratory confirmed pathogens, comorbidities using clinical signs and symptoms, and food supplementation (Table 3). The 15 PMTCT articles found addressed elimination of paediatric HIV infections, transmission of HIV to the babies, and improvement of survival in infected mothers and their exposed children.

**Table 3:** Studies utilising routinely collected data on HIV treatment and retention in care in Zambia

First author	Data collection period	Sample size	Sampled population	Outcomes assessed
Cantrell et al. [52]	2004–2005	636	Adults on ART	Adherence to medication, CD4, weight gain
Koethe et al. [53]	2004–2008	27,915	Adults initiating ART	Weight gain, death, treatment failure, BMI
Tirivayi et al. [54]	2009	291	Adults on ART	Adherence to medication, CD4, BMI
Koethe et al. [55]	2004–2009	56,612	Adults on ART	CD4, mortality, BMI
Stringer et al. [56]	2004–2007	14,736	Adults on ART	Single dose substitution, CD4 count, haemoglobin level, BMI, mortality
Chi et al. [57]	2007–2010	18,866	Adults initiating ART	CD4, clinical disease staging, BMI, serum creatinine adherence, mortality
Chi et al. [58]	2007–2009	10,485	Adults on ART	Drug substitution, mortality, loss to follow-up, withdrawal and death
Chi et al. [59]	2004–2008	24,366	Adults on ART	CD4 counts, age clinical staging, haemoglobin, tuberculosis co-infection, adherence
Chi et al. [60]	2007	33,704	Adults on ART	Cut-off points defining loss to follow-up, sensitivity, specificity, misclassification rate
Giganti et al. [61]	2004–2010	40,410	Adults on ART	Haemoglobin level, CD4, ART regimen
Vinikoor et al. [62]	2015	20,308	Adults on ART	HBsAg, CD4, BMI, WHO staging
Mulenga et al. [63]	2004–2007	25,779	Adults on ART	Mortality, creatinine clearance
Stringer et al. [64]	2004–2005	21,755	Adults initiating ART	Clinical staging, CD4, mortality, BMI, haemoglobin level, adherence
Seu et al. [65]	2009–2012	68	Adults failing treatment	CD4 count, adherence, HIV drug resistance mutations
Krebs et al. [66]	2005	1343	Adults lost to follow-up	CD4 count, BMI, mortality, home visit categories (traced, untraceable, died)
Vinikoor et al. [67]	2004–2010	53,015	Adults missing pharmacy refills	CD4 count, clinical staging, pharmacy refills, adherence, ART regimen
Vinikoor et al. [68]	2004–2011	92,130	Adults on ART	Adherence, CD4 count, mortality, long-term follow-up
Harris et al. [69]	2005–2007	20,153	Tuberculosis/HIV co-infected adults	Enrolment on ART, CD4 count, WHO staging
Mweemba et al. [70]	2011–2013	91,130	Adults initiating ART	Hepatitis B co-infection, WHO staging, CD4 count
Deo et al. [71]	2007	13	Laboratories	CD4 count, haemoglobin, liver function test
Chi et al. [72]	2004–2006	6740	Women exposed to nevirapine	WHO stage, CD4 cell count, status, BMI
Bolton-Moore et al. [73]	2004–2007	4975	Children on ART	CD4 percentage, weight-for-age Z scores, clinical staging, haemoglobin level, mortality
Mubiana‐Mbewe et al. [74]	2004–2006	1705	Children enrolled into care	CD4 percentage, clinical staging, haemoglobin level
Scott et al. [75]	2006–2011	1334	Children on ART	CD4 percentage, fixed and variable unit costs
Kiage et al. [76]	2009–2011	822	Mother-infant pairs	WHO staging, CD4/CD8 percentage, HIV, haemoglobin panel, maternal-CD4 count
Sutcliffe et al. [77]	2004–2008	1278	Children on ART	Enrolment in ART, loss to follow-up, mortality, clinical staging, CD4 percentage
Iyer et al. [78]	2006–2011	1102	Children initiating ART	Age, CD4 percentage, ART initiation, full blood count, blood chemistry
Sutcliffe et al. [79]	2004–2008	863	Children on ART	Mortality, CD4, HIV, haemoglobin level
Sutcliffe [80]	2000–2002	492	Children on ART	CD4 count, haemoglobin level, mortality
Van Dijk et al. [81]	2007–2012	77	Children on ART	Weight-for-age Z scores, CD4 percentage
Van Dijk et al. [82]	2007–2010	198	Children on ART	Treatment outcomes, viral load, CD4 percentage, retention in care, mortality
Sutcliffe et al. [83]	2007–2009	193	Children initiating ART	Weight-for-age and height-for-age Z scores
Nkamba [84]	Not stated	59	Children on ART	T cell subsets CD4 and CD8 memory
Van Dijk et al. [85]	2007–2008	192	Children on ART	Years of receiving ART, distance from clinic, CD4 percentage, weight-for-age Z score
Sinkala et al. [86]	2005–2006	5609	Adults only	Colonoscopy, laparoscopy, culture results
Sheyo [87]	2009–2010	452	Adults and children	HIV status, burn history, burn outcome and management
Brugha et al. [88]	2004–2007	39	Health facilities	VCT, ART, PMTCT, childhood immunisation service and coverage trends
Kancheya et al. [89]	2003–2006	203	Adults	HIV status, VCT
Kaile et al. [90]	2004	18	Adults on ART	Blood pressure serum potassium, creatinine and sodium, Karnofsky score, WHO staging

*ART* antiretroviral therapy, *BMI* body mass index, *PMTCT* prevention of mother-to-child transmission, *VCT* voluntary counselling and testing

We found four articles on HTC covering couple counselling and provider-initiated testing and counselling. Articles on social and behaviour change looked at creating demand for adherence, prevention interventions, improved biomarkers and treatment uptake. Treatment and care had the largest number of articles with 39 articles covering the topic (Table 3). Of the 62 articles, 33 full length papers utilised adult only routinely collected data and addressed retention in care, access to HIV treatment, mortality and clinical outcomes. A total of 16 full-length articles used data from only women, covering contraception, PMTCT and antenatal HIV prevalence rates. The articles using data on women only were published between 2010 and 2011, and had sample sizes ranging from 1435 to 138,884. There were 13 peer-reviewed articles that addressed paediatric HIV care and treatment. These were published between 2007 and 2013, with sample sizes ranging from 1120 to 4975. Our search did not reveal any articles utilising routinely collected HIV data specifically on adolescents aged 10–24 years old.

**Fig. 2:**
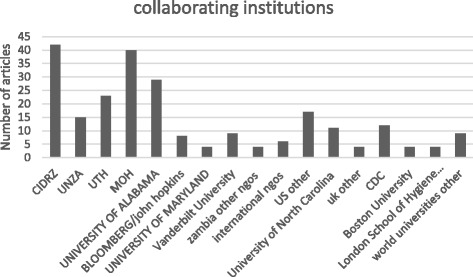
Graph showing number of articles published by each collaborating institution

The 62 papers analysed were published in collaboration with partner institutions (Fig. 2). The Centre for Infectious Disease Research in Zambia and the University of Alabama had the highest contribution, with collaboration on 42 and 29 papers, respectively. The staff from national and district levels of the Ministry of Health participated in 40 of the published articles, while the lower collaboration was from institutions based in the United Kingdom, with collaboration on only 8 articles, four from LSHTM and four from other universities.

## Discussion

The review of published articles showed that considerable strides are being made in utilisation of routinely collected HIV data in Zambia. A total of 62 articles were found and considered in this review. Treatment and retention in care and PMTCT had the highest contribution, with counselling and social and behavioural change having four articles each. However, we could not find published papers that utilised quantitative data analysis methods in our search on VMMC and condom programming despite the importance of these programmes and the inclusion of data from these programmes in SmartCare. The broad focus of the literature search on HIV in Zambia should have identified many papers on condom programming or VMMC, but the only papers found were qualitative studies on these topics. It was also observable during the search process that quantitatively analysed studies on HIV in adolescents in the country were limited and information for this age group has to be extracted from paediatric and adult studies.

This study was a collection of articles covering a diverse range of topics, which meant that no meta-analysis of the studies was possible. One of the goals of this paper was to highlight the range and diversity of the topics available for analysis using routinely collected data, and to explore the gaps in the published literature so far. The main topics were grouped into treatment and retention in care, PMTCT, HTC, condom programming and VMMC. Our search on VMMC and condom programming revealed no quantitatively analysed papers and few qualitatively analysed papers.

We did not include a large number of qualitatively analysed, clinical trial and survey-based articles, which have made important contributions to policy change in HIV care and treatment in Zambia. Further, risk of bias in individual study papers selected was not prioritised during the selection process since the rationale of the review was to assess the extent of utilisation of routinely collected data in the country. Only peer-reviewed articles where included because the assumption was that the peer-review process implies some form of quality control for biases in selected papers. In addition, only one of the authors reviewed the titles and abstracts, which could be a source of bias. However, as far as we are aware, this is the first study to provide such a baseline of studies for future referral.

The total number of published articles found in our literature search on the six HIV programmatic areas using routinely collected data meeting our criteria was quite low (an average of six articles per year) considering that these have been published in the past 10 years. This finding is lower than the 32 articles per year reported by Uthman [17], but in line with findings by the Ministry of Health [18], where the use and analysis of routinely collected data were found to be inadequate in Zambia, with analysed data displayed in graphs and information from the districts rarely used for decision-making at district levels. The reasons for these low numbers could be the limited data analysis skills, unavailability of data analysis software, disapproval or lack of support from supervisors, and lack of time and opportunity [9–11, 18–20]. It could be further argued that the limited use of routinely collected data was due to lack of knowledge on the benefits of analyzing such data at facility and district levels and poor data management, which could be alleviated by deliberate policy from government to support existing staff capacity building, operational research and publication of findings [18, 21].

Treatment and retention in care had the largest number of studies. This is in line with global trends in HIV prevention strategies where treatment and retention in care have been identified as the most effective HIV prevention tool among the biomedical prevention tools analysed to date [22, 23]; more research is encouraged in these areas. However, considering the period under review, the number of studies found on retention and care were rather low. Similar trends of low levels of publication in this area have been attributed to long follow-up periods required to monitor retention in care as well as to inconsistent information systems that make it difficult to track patients that seek care from multiple facilities [24].

There was also a limited number of studies that looked at children born with HIV infection identified in our search. This is in line with findings from a systematic review of care and retention in HIV-infected children in low- and middle-income countries, where limited data were also found in Asia, Eastern Europe and Latin America [25], attributed to emphasis on studies on adult data. It was also apparent that no quantitative peer-reviewed studies on treatment and retention among adolescents already in HIV care in Zambia were found in our search. Data on this age group has to be extracted from paediatric and adult studies. Similar findings have been reported in studies conducted in southern Africa in 2009 [26] and 2010 [27], where few data were reported on perinatally infected adolescents with most of the available data on adherence and outcomes emerging from the developed world. It is further argued that data for adolescents in southern Africa are disaggregated into 0–14, 15–19 and 15–24 year age groups, which makes it difficult to ascertain adolescent-specific data since, in most cases, the data includes very young children or adults [28].

The search on condom programming revealed mostly intervention studies in settings where prospective users could access them. Reasons for this could be the mode of distribution, which is restricted to public health facilities and to private health facilities only on request [29]. Similarly, Kane et al. [30] argued that use of aggregate data on condom sales does not provide information on utilisation of condoms after they are obtained, resulting in the need for in-depth analysis on factors associated with condom use. The same could be concluded on usefulness of aggregate condom distribution data. Moreover, condom distribution data are difficult to document in routinely collected data and thus there is heavy reliance on survey data [7]. There is an urgent need to understand the demographics of condom distribution in the country. Similar trends were revealed in the VMMC programme, which also yielded low numbers of peer-reviewed articles that utilised routinely collected quantitative data, despite the country not meeting its circumcision targets [7].

## Conclusion

There are positive advances being made in the HIV programme in the use of routinely collected data in Zambia. This progress must be nurtured and enhanced if Zambia is to reach elimination stages in HIV control. However, more efforts must be put into research and publishing results in critical areas, such as paediatric and adolescent care, VMMC and condom distribution, in order to build the skills and knowledge-base to deliver HIV services. Research on adolescent and childhood HIV morbidity and mortality outcomes as well as social behavioural change needs is important because HIV-infected adolescents and children are the key population in reducing HIV spread in their generation.

To improve the use of routinely collected data for use in publications the government could deliberately put in place policies to prioritise training of civil servants working in various programmes in operational research and consequently fund publishing of findings. These published articles would aid in international resource mobilisation for most programmes in the country as programme level data can be easily accessed in peer-reviewed articles.

## Abbreviations

HTC: HIV testing and counselling
PMS: patient monitoring system
PMTCT: prevention of mother to child transmission
VMMC: voluntary male circumcision
